# Autophagy gene-dependent intracellular immunity triggered by interferon-γ

**DOI:** 10.1128/mbio.02332-23

**Published:** 2023-10-31

**Authors:** Michael R. McAllaster, Jaya Bhushan, Dale R. Balce, Anthony Orvedahl, Arnold Park, Seungmin Hwang, Meagan E. Sullender, L. David Sibley, Herbert W. Virgin

**Affiliations:** 1 Department of Molecular Microbiology, Washington University School of Medicine, St. Louis, Missouri, USA; 2 Vir Biotechnology, San Francisco, California, USA; 3 Department of Pathology and Immunology, Washington University School of Medicine, St. Louis, Missouri, USA; 4 Department of Pediatrics, Washington University School of Medicine, St. Louis, Missouri, USA; 5 Division of Infectious Diseases, Department of Medicine, Edison Family Center for Genome Sciences & Systems Biology, Washington University School of Medicine, St. Louis, Missouri, USA; 6 Department of Internal Medicine, UT Southwestern Medical Center, Dallas, Texas, USA; Max Planck Institute for Infection Biology, Berlin, Germany; University of Massachusetts Medical School, Worcester, Massachusetts, USA

**Keywords:** interferons, *Toxoplasmsa gondii*, autophagy, UFMylation, norovirus

## Abstract

Genes required for the lysosomal degradation pathway of autophagy play key roles in topologically distinct and physiologically important cellular processes. Some functions of *ATG* genes are independent of their role in degradative autophagy. One of the first described of these *ATG* gene-dependent, but degradative autophagy independent, processes is the requirement for a subset of *ATG* genes in interferon-γ (IFNγ)-induced inhibition of norovirus and *Toxoplasma gondii* replication. Herein, we identified additional genes that are required for, or that negatively regulate, this innate immune effector pathway. Enzymes in the UFMylation pathway negatively regulated IFNγ-induced inhibition of norovirus replication via effects of *Ern1*. IFNγ-induced inhibition of norovirus replication required *Gate-16* (also termed *GabarapL2*), *Wipi2b*, *Atg9a*, *Cul3*, and *Klhl9* but not *Becn1* (encoding Beclin 1), *Atg14*, *Uvrag*, or *Sqstm1*. The phosphatidylinositol-3-phosphate and ATG16L1-binding domains of WIPI2B, as well as the ATG5-binding domain of ATG16L1, were required for IFNγ-induced inhibition of norovirus replication. Other members of the *Cul3*, *Atg8*, and *Wipi2* gene families were not required, demonstrating exquisite specificity within these gene families for participation in IFNγ action. The generality of some aspects of this mechanism was demonstrated by a role for GATE-16 and WIPI2 in IFNγ-induced control of *Toxoplasma gondii* infection in human cells. These studies further delineate the genes and mechanisms of an *ATG* gene-dependent programmable form of cytokine-induced innate intracellular immunity.

**IMPORTANCE:**

Interferon-γ (IFNγ) is a critical mediator of cell-intrinsic immunity to intracellular pathogens. Understanding the complex cellular mechanisms supporting robust interferon-γ-induced host defenses could aid in developing new therapeutics to treat infections. Here, we examined the impact of autophagy genes in the interferon-γ-induced host response. We demonstrate that genes within the autophagy pathway including *Wipi2*, *Atg9*, and *Gate-16*, as well as ubiquitin ligase complex genes *Cul3* and *Klhl9* are required for IFNγ-induced inhibition of murine norovirus (norovirus hereinafter) replication in mouse cells. *WIPI2* and *GATE-16* were also required for IFNγ-mediated restriction of parasite growth within the *Toxoplasma gondii* parasitophorous vacuole in human cells. Furthermore, we found that perturbation of UFMylation pathway components led to more robust IFNγ-induced inhibition of norovirus via regulation of endoplasmic reticulum (ER) stress. Enhancing or inhibiting these dynamic cellular components could serve as a strategy to control intracellular pathogens and maintain an effective immune response.

## INTRODUCTION

Macroautophagy (autophagy herein) requires formation of an isolation membrane that envelops cytoplasmic materials, organelles, or invading pathogens in a closed double membrane-bound autophagosome. Autophagosomes fuse with lysosomes to facilitate degradation of captured material (1, 2). Autophagy requires a series of autophagy genes (*ATG* genes), many of which are conserved broadly in evolution. It is now clear that these essential *ATG* genes are also required for additional topologically distinct cellular processes that have significant physiologic importance (3–5). Many of these autophagy-independent, but *ATG* gene-dependent, processes occur in myeloid cells. These include essential roles for *ATG* genes in the anti-microbial action of the key cytokine interferon-γ (IFNγ) (also referred to as type II IFN), the deposition of the ATG8-family proteins on the cytoplasmic surface of phagosomes (2, 6), the fusion of lysosomes to the polarized ruffled membrane of osteoclasts to secrete lysosomal proteases for extracellular degradation of bone (7), the regulation of neutrophilic inflammation during *Mycobacterium tuberculosis* infection (8), and the inhibition of inflammatory activation of tissue-resident macrophages (9). Of particular note, the role of *ATG* genes in the actions of IFNγ is fundamentally important to the survival of mammals because this cytokine plays an essential role in triggering cell-intrinsic immunity to intracellular bacteria, viruses, and parasites.

The fact that *ATG* genes play roles in both autophagy and topologically distinct cellular processes makes it imperative to further define the molecular machinery that is in common to, or distinguishes, these distinct cellular events (10). Here, we focus on IFNγ-induced *ATG* gene-dependent intracellular immunity to murine norovirus (norovirus herein) and *Toxoplasma gondii* (*T. gondii*). Inhibition of intracellular replication of these pathogens requires *ATG* genes *Atg7*, *Atg5*, *Atg16l1*, *Atg12*, *Atg3*, and *Atg8* family members (11–19). ATG3, ATG7, and the ATG5-ATG12-ATG16L1 (herein the ATG5-12-16L1 complex) are required for the ubiquitin-like conjugation system of autophagy that conjugates phosphatidyl-ethanolamine (PE) to ATG8 proteins to localize them on membranes (1, 4). However, the topology of the intracellular events triggered by IFNγ is distinct for the two pathogens. *T. gondii* replicates in a parasitophorous vacuole (PV) sequestered away from the cytoplasm, while norovirus replicates on the cytoplasmic face of membranes. In mice, IFNγ-induced *ATG* gene-dependent clearance of *T. gondii* occurs by destruction of the parasitophorous vacuole (11, 14), while in human cells this pathway limits replication but may not eliminate the parasite (16, 20). In contrast, IFNγ inhibits norovirus replication by disrupting the formation and/or function of the membranous replication compartment upon which viral RNA synthesis occurs (12, 17).

Here, we show that an immortalized murine macrophage-like microglial cell line (BV-2 cells) (21) recapitulates *ATG* gene-dependent events in IFNγ-induced inhibition of norovirus replication as originally described in primary macrophages. We then identified additional positive and negative regulators of this process. *Ufc1* and *Uba5* negatively regulated IFNγ inhibition of norovirus replication. In contrast, *Atg9a*, *Wipi2*, *Gate-16*, *Cul3*, and *Klhl9* were required for efficient IFNγ-induced inhibition of norovirus replication. The ATG16L1- and phosphatidylinositol 3-phosphate [PtdIns(3)P]-binding domains of WIPI2B, as well as the ATG5-binding domain of ATL16L1, were required for efficient IFNγ-induced inhibition of norovirus replication. *WIPI2* and *GATE-16* were also required for IFNγ-induced inhibition of *T. gondii* replication in human cells. In contrast to findings in the norovirus system, *SQSTM1* (herein *P62*) was required for IFNγ-induced inhibition of *T. gondii* replication, but not for control of replication of norovirus in murine cells. Thus, components of the autophagy machinery outside of the ubiquitin-like conjugation systems involving the ATG5-12-16L1 complex are important components of a cellular immune mechanism used by IFNγ to block intracellular pathogen replication, potentially identifying targets for modulation of this type of immunity. The selective requirement for *Gate-16*, *Wipi2*, *Cul3*, *Atg9a*, and *Klhl9*, but not other members of their respective families, demonstrates the specificity of this form of intracellular immunity.

## RESULTS

### IFNγ inhibits norovirus replication in BV-2 cells in a *Stat1*- and *Irf1*-dependent manner

**Fig 1:**
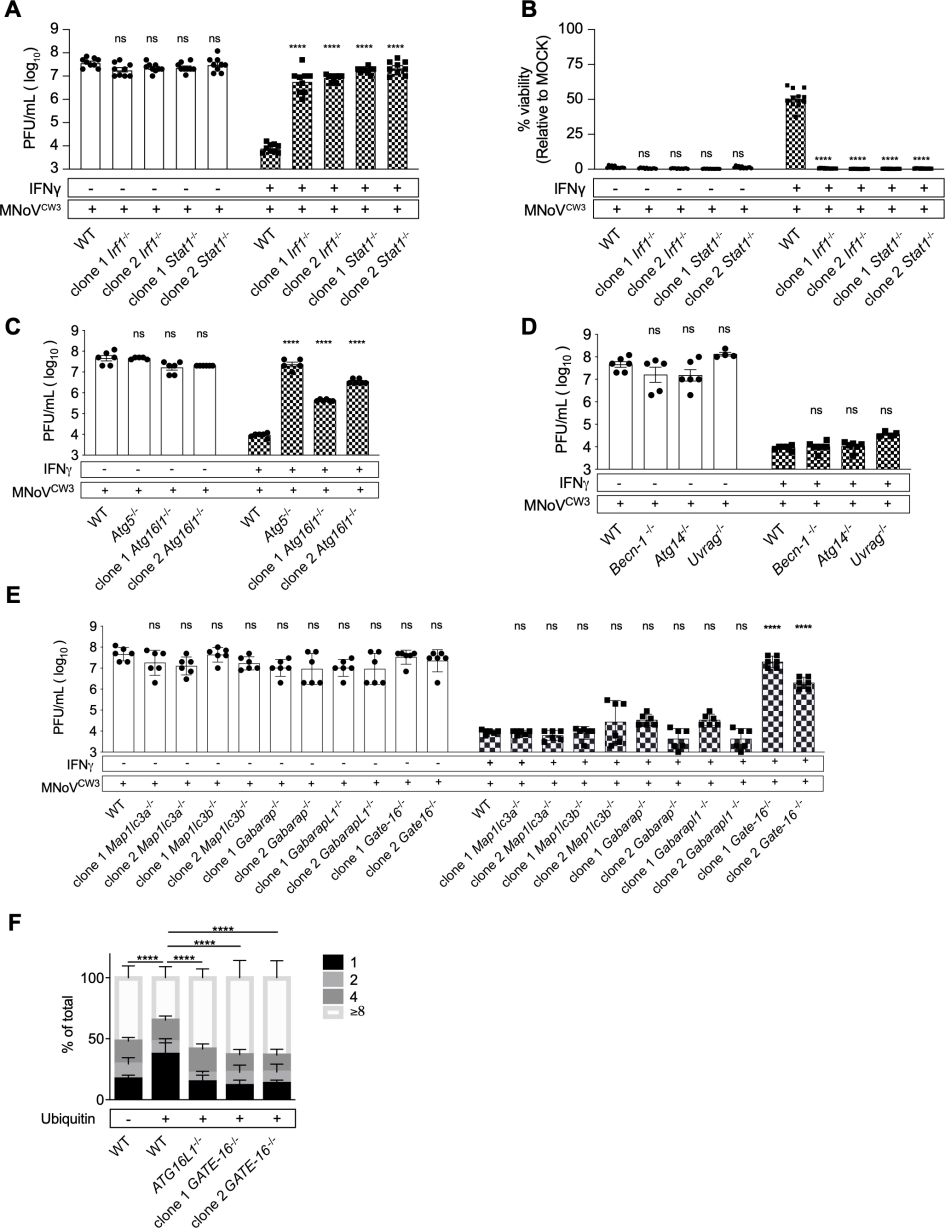
*Stat1*, *Irf1 Atg5*, *Atg16l1*, and *Gate-16* are required for IFNγ-induced inhibition of norovirus replication in BV-2 cells, *Atg14*, *Becn1*, and *Uvrag* are not; *GATE-16* is required for IFNγ-induced growth restriction of *T. gondii* in HeLa cells. (A) Plaque assay of WT, *Irf1*^−/−^, and *Stat1*^−/−^ BV-2 cells as described in Fig. S1. (B) Viability assay of WT, *Irf1*^−/−^, and *Stat1*^−/−^ BV-2 cells as described in Fig. S1. (C–E) Plaque assay of WT, *Atg5*^−/−^, *Ag16l1*^−/−^, *Atg14*^−/−^, *Becn1*^−/−^, *Uvrag*^−/−^, and *Atg8* family members in BV-2 cells. (F) *WT*, *ATG16L1^−/−^*, and GATE16^−/−^ were treated with IFNγ and infected with *T. gondii*. Parasites within ubiquitin-positive (+) and ubiquitin-negative (−) vacuoles were counted by immunofluorescence microscopy. Average data pooled from 2 to 3 independent experiments are represented as means ± standard error of the mean (SEM). *P ≤ 0.05, **P ≤ 0.01, ***P ≤ 0.001, and ****P ≤ 0.0001 were considered statistically significant. ns, not significant. *P* value was determined by two-way analysis of variance (ANOVA) with Dunnett’s multiple comparison test; for *T. gondii* assay, *P* value was determined by two-way ANOVA with Tukey’s multiple comparison test.

BV-2 cells are permissive for replication of murine norovirus strain MNoV^CW3^ (22), allowing us to test whether, in these cells, norovirus replication was inhibited by recombinant IFNγ as measured by plaque assay and cellular ATP content as a proxy for cell viability (Fig. S1). Pretreatment of BV-2 cells with IFNγ decreased MNoV^CW3^ (norovirus hereinafter) replication ~5,000-fold (Fig. 1A) and cytopathicity by 50% (Fig. 1B). *Stat1* and *Irf1* encode transcription factors essential for IFNγ responses (23) and are required for robust IFNγ-induced inhibition of norovirus replication both *in vitro* in primary macrophages and *in vivo* (24–26). To determine whether these transcription factors were required for IFNγ-induced inhibition of norovirus replication in BV-2 cells we generated two independent clonal *Stat1* or *Irf1* knockout BV-2 cell lines (*Stat1^−/−^*, *Irf1^−/−^*) (Fig. 1A and B). Throughout this work, we confirmed deletion of genes in cell lines using next generation sequencing (Table S1). IFNγ failed to efficiently inhibit norovirus replication and cytopathicity in either *Stat1^−/−^* or *Irf1^−/−^* cells (Fig. 1A and B), faithfully replicating the requirement for these genes in primary cells (26).

### The ATG5-12-16L1 complex is required for IFNγ-induced inhibition of norovirus replication in BV-2 cells

The ATG5-12-16L1 complex is crucial for IFNγ-induced control of norovirus in primary bone marrow-derived murine macrophages (12). Using *Atg5* knockout BV-2 cells (*Atg5*^−/−^) (27, 28), we confirmed that *Atg5* was required for IFNγ-induced inhibition of norovirus replication (Fig. 1C). To further define the role of the ATG5-12-16L1 complex we generated two independent clonal knockout BV-2 cell lines for *Atg16l1* (*Atg16l1^−/−^*) (Table S1) (27). IFNγ failed to efficiently inhibit norovirus replication in cells lacking *Atg16l1* (Fig. 1C). Deep sequencing confirmed saturating indel frequencies in *Atg16l1* clonal knockout cells (Table S1) supporting a role for *Atg16l1* in IFNγ inhibition of norovirus replication in these cells.

### The *ATG* genes *Becn1*, *Atg14*, and *Uvrag* are not required for IFNγ-induced inhibition of norovirus replication

Certain upstream components of the autophagy pathway are not required for IFNγ-induced inhibition of norovirus or *T. gondii* replication (11, 12, 16, 17). To confirm these observations in BV-2 cells, we determined whether IFNγ efficiently inhibits norovirus replication in clonal *Atg14*^−/−^ BV-2 cells (27, 28) and clonal *Becn1*^−/−^ cell lines (28) (Fig. S3). *Atg14*, *Becn1*, and *Uvrag* were not required for IFNγ-induced inhibition of norovirus replication (Fig. 1D). We sought to extend these findings using cells lacking the VPS34 kinase which is responsible for PI3P generation by these Class II PI3 kinase complexes, but were unable to isolate viable cells lacking this protein. Additionally, in the setting of viral infection and IFNγ treatment, the drugs wortmannin (29), apilimod (30), and alpelisib (31) proved too toxic to provide interpretable data related to phosphatidylinositol involvement (not shown). A recent report using murine embryonic fibroblasts depleted for VPS34 expression found no evidence of a role for VPS34 in this form of intracellular immunity (32). Together with the data on the role of *Stat1*, *Irf1*, *Atg5*, and *Atg16l1*, these data support the validity of BV-2 cells as a model to further define mechanisms of IFNγ-induced *ATG* gene-dependent immunity to norovirus and indicate that the Class III PI3 kinase complexes that include Beclin 1 together with either ATG14 or UVRAG and that are required for degradative autophagy, are not required for this type of intracellular immunity.

### Role of Atg8 family members in IFNγ-induced inhibition of norovirus and *T. gondii* infection

The ATG8 family of proteins plays a key role in multiple aspects of autophagy and *ATG* gene functions that are independent of degradative autophagy. Evidence has accumulated that individual family members can provide specific functions in different cell biology processes (18, 33). We therefore isolated two independent clones of cells lacking ATG8 family genes *Map1lc3a*, *Map1lc3b*, *Gabarap*, *GabarapL1*, or *Gate-16*. Only *Gate-16* was required for IFNγ-induced inhibition of norovirus replication (Fig. 1E).

To assess the generality of this finding across pathogens, we analyzed the growth of *T. gondii* in the parasitophorous vacuole of human cells, using *ATG16L1*-deficient cells as a control for a gene known to be required for the activity of IFNγ against this pathogen (16). We quantitated *T. gondii* replication within the PV in parental and two independent *GATE-16^−/−^* human HeLa cells (Table S1), using a type III parasite that is susceptible to *ATG* gene-dependent IFNγ-induced growth control (16, 20). *T. gondii* multiplies by binary fission with a half-life of ~8 hours, generating vacuoles containing clusters of 1 to ≥8 parasites over 24 hours. A subset of parasitophorous vacuoles becomes labeled by ubiquitin in IFNγ-treated cells (16). Ubiquitin-positive vacuoles are targeted in an *ATG* gene-dependent manner that impairs replication resulting in decreased numbers of parasites per vacuole (16). We therefore counted parasites per ubiquitin-positive vacuole in wild type and *GATE-16^−/−^* HeLa cells (Fig. 1F) (16). In wild-type cells, we did not observe a growth restriction phenotype in PVs lacking ubiquitin (Fig. 1F, left); however, most ubiquitin-positive vacuoles contained only one parasite, indicative of IFNγ-induced growth restriction (Fig. 1F). In the absence of either *ATG16L1* [as previously reported, (14, 16)] or *GATE-16*, IFNγ failed to efficiently restrict parasite growth, and fewer PVs containing one parasite were observed while the majority containing ≥8 parasites (Fig. 1F). Thus, the role of *GATE-16* in IFNγ-induced inhibition of intracellular replication extends from a cytoplasmic RNA virus to an intravacuolar apicomplexan parasite.

### CRISPR screen for identification of genes involved in IFNγ-induced inhibition of norovirus cytopathicity in BV-2 cells

**Fig 2:**
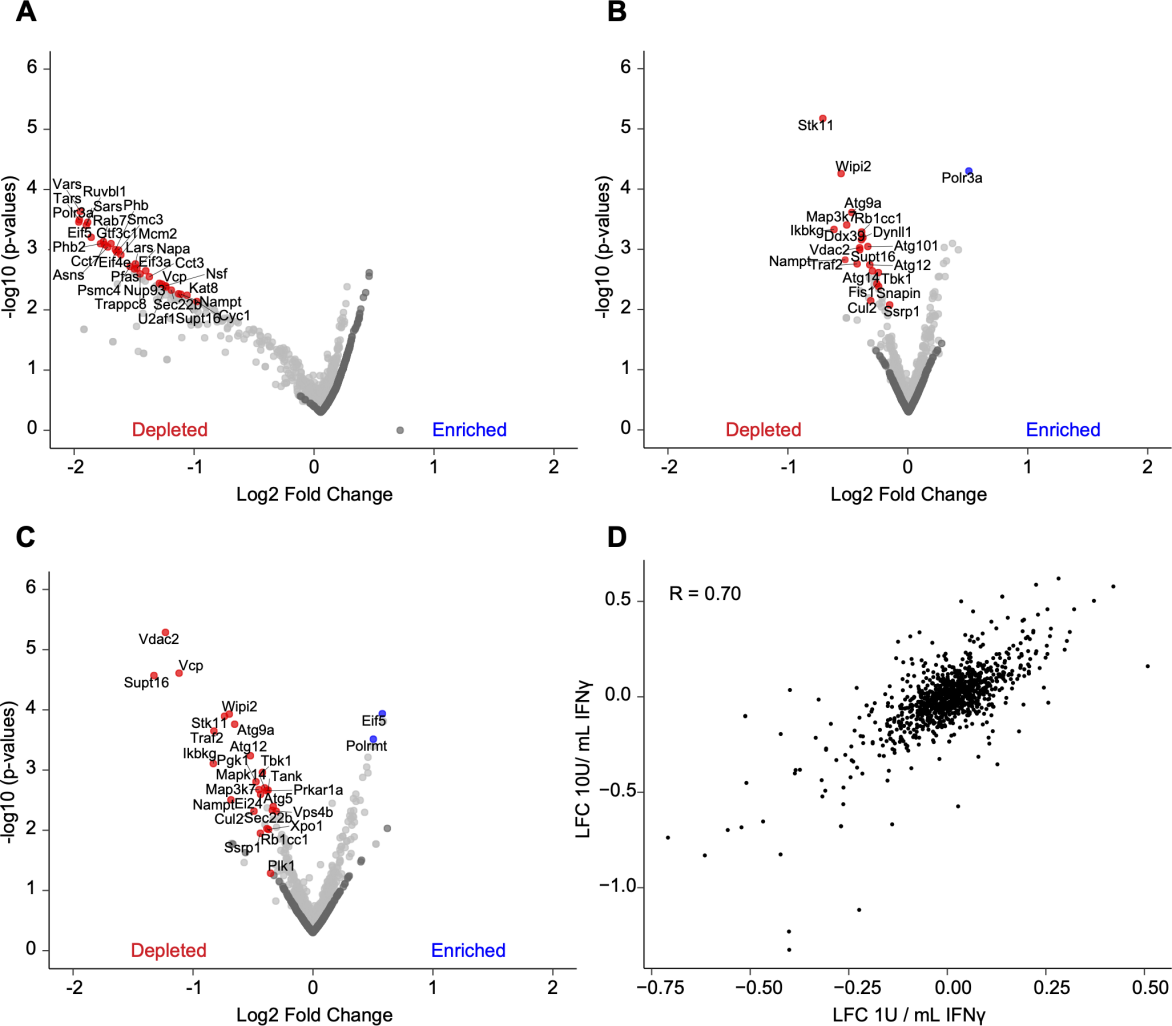
Identification of genes required for viability in passage or IFNγ-treated BV-2 cells. (A) Volcano plot of mock treatment guides enriched or depleted relative to 5 days post-puromycin selection. (B) Volcano plot of guides enriched or depleted after 1 U/mL IFNγ treatment relative to mock treatment. (C) As in (B) for 10 U/mL IFNγ treatment. (D) Average log2 fold change (LFC) of 1 U/mL IFNγ condition versus LFC of 10 U/mL IFNγ condition. Pearson correlations are indicated. For volcano plots, the LFC of all sgRNAs for each gene is plotted against the −log10(*P* value) for each gene. Blue and red highlighted genes in (A–C) represent a STARS score with FDR < 0.01 and dark gray genes represent non-targeting guides.

We used CRISPR screening to identify candidate genes for a role in IFNγ action (Fig. S2). We designed an autophagy sgRNA library containing 1–4 independent guides targeting 695 candidate genes (Table S2). We included 300 guides with no known target as controls (2,979 guides total) (34). Genes were selected using three criteria: (i) present in the autophagy interaction landscape, including the baits used, as defined by Behrends et al. (35); (ii) murine genes related to autophagy using gene ontology (GO) (36, 37); and (iii) murine genes corresponding to human genes related to autophagy using GO (36, 37). The gene encoding CAPRIN1, recently shown to have a role in this form of immunity, was not included in the CRISPR screen (32). To enhance the sensitivity of this CRISPR screen, we targeted the generation of a cell pool with each guide represented by ~2,000 cells (38). After experimental selection (Fig. 2A), we quantified guides by DNA sequencing and determined the significance of differences in guide frequencies in different comparisons using STARS and negative binomial analysis (22, 27, 28, 34, 39) with a threshold of false discovery rate (FDR) < 0.1 to identify genes for further consideration (28).

### Genes required for cell survival in the absence of norovirus infection

The effects of genes required for cell survival or proliferation may obscure the identification of those required for a phenotype, especially when cell death is part of the biology being studied as is the case for norovirus replication and IFNγ treatment (22, 28, 34). We, therefore, identified differences in guide frequency between the original cell library and cells passaged under mock conditions (Fig. 2A; Table S3) as well as between cells passaged under mock conditions compared to those treated with IFNγ at two doses (Fig. 2B and C; Table S4). We analyzed both 1 U/mL and 10 U/mL because the lower dose is non-toxic, while the higher dose alters proliferation and/or cell survival of wild-type BV-2 cells (28, 34). The mock passage did not enrich guides for any gene but decreased guides for 52 genes, suggesting that these are essential genes for the growth or survival of BV-2 cells under these conditions. Among the targeted genes were *Cdc37*, *Adsl*, and *Cct2*. Mutations in ADSL result in the rare autosomal recessive disorder adenylosuccinate lyase deficiency (40), while CCT2 mutations evoke the rare disease Leber congenital amaurosis (41). We compared these candidate essential genes to those identified in Project Achilles (42–44). Three of 52 genes lacked a human homolog and one did not have specific guides in Project Achilles. Therefore, 48 candidate essential genes were considered for this comparison (Table S3). Forty-three of 48 (95%) depleted genes were considered common essential among immortalized cells (Table S3) (42–44), thus validating our screening approach via deprioritizing analysis of genes whose guides were depleted under control conditions.

### Identification of genes enriched or depleted in the presence IFNγ

IFNγ treatment at 1 U/mL enriched guides for seven genes (Fig. 2B; Table S4). Escalation of the IFNγ dose to 10 U/mL enriched guides for 20 genes (Fig. 2C; Table S4). Among the targeted genes were *Eif5*, *Polrmt*, *Eif3a*, *Polr3a*, *Eif4g2*, and *Eif4e* (Fig. 2B and C; Table S4). These genes regulate mRNA translation and immune cell activation (45–47). IFNγ treatment at 1 U/mL depleted guides for 25 genes (Fig. 2B; Table S4), and at a dose of 10 U/mL depleted guides for 24 genes (Fig. 2C; Table S4). We observed agreement at the gene level between treatment with 1 U/mL or 10 U/mL IFNγ with a Pearson’s correlation of 0.70 (Fig. 2D). Among the targeted genes were *Traf2*, *Tbk1*, *Ikbkg*, *Tank*, *Ei24*, *Map3k7*, *Atg5*, and *Atg14* (Fig. 2B and C; Table S4). *Traf2*, *Tbk1*, *Ikbkg*, *Tank*, *Ei24*, and *Map3k7* regulate the tumor necrosis factor receptor signaling pathway (48–50). Consistent with these results, IFNγ-induced cell death in BV-2 cells is mediated by tumor necrosis factor (28). Our results confirmed the critical role of *Atg14* and *Atg5* in inhibiting IFNγ-induced cell death in BV-2 cells (Fig. 2B; Table S4) (28). Guides for *Wipi2*, *Rb1cc1* (also known as *Fip200*), *Atg9a*, *Atg101*, and *Atg12* were also depleted, though these genes were not reported to protect BV-2 cells from IFNγ-induced cell death (Fig. 2B and C; Table S4) (28).

### Identification of genes that enhance IFNγ-induced inhibition of norovirus-induced cytopathicity

**Fig 3:**
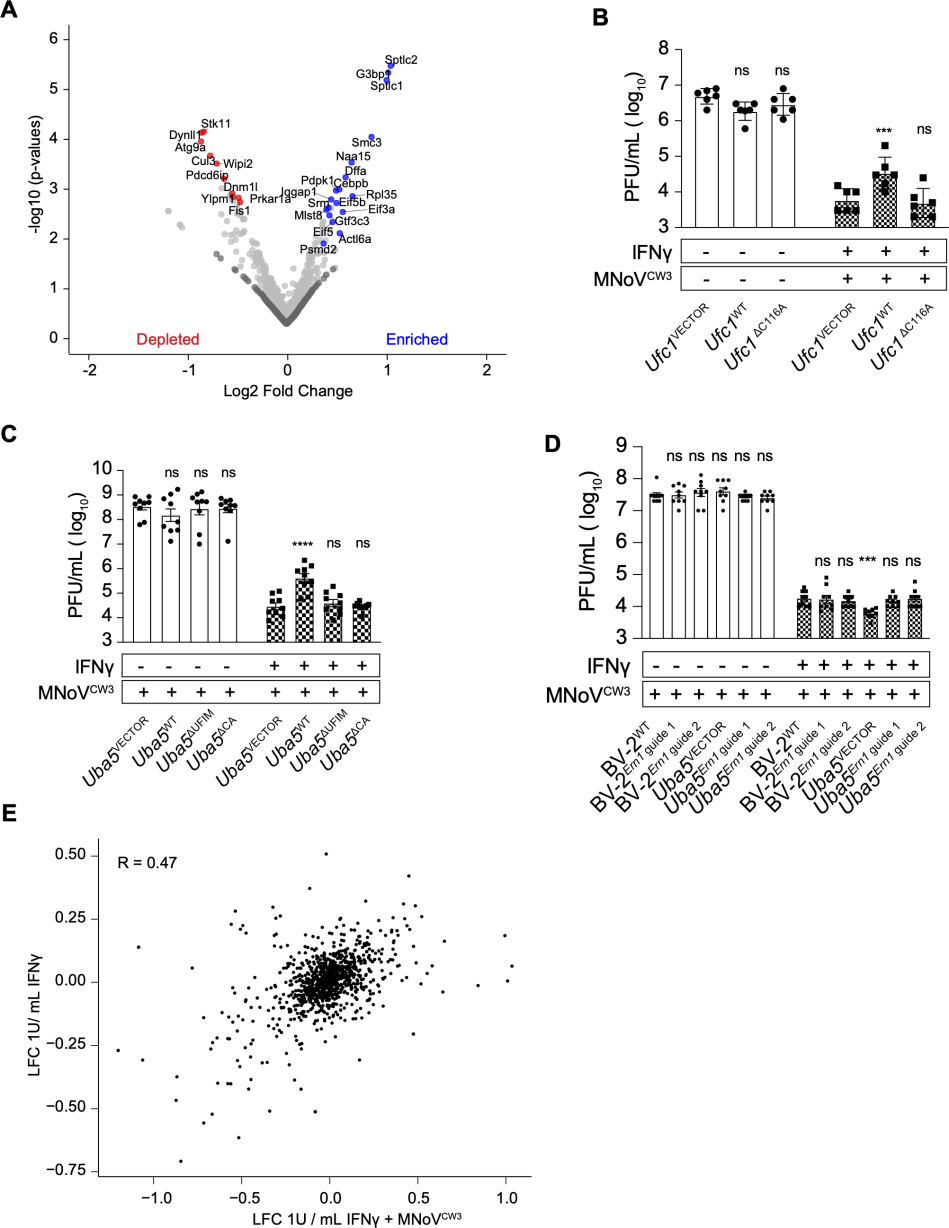
Identification of genes required for IFNγ-induced norovirus cytopathicity in BV-2 cells. (A) Volcano plot of guides enriched or depleted after 1 U/mL IFNγ + norovirus infection relative to mock treatment. (B) Plaque assay of *Ufc1*^VECTOR^, *Ufc1*^WT^, and *Ufc1*^ΔC116A^ BV-2 cells as described in Fig. S1. (C) Plaque assay of *Uba5*^VECTOR^, *Uba5*^WT^, *Uba5*^ΔUFIM^, and *Uba5*^ΔCA^ BV-2 cells as described in Fig. S1. (D) Plaque assay of BV-2^WT^, BV-2^Ern1 guide 1^, BV-2^Ern1 guide 2^, *Uba5*^VECTOR^, *Uba5*^Ern1 guide 1^, and *Uba5*^Ern1 guide 2^ cells as described in Fig. S1. (E) Average LFC of 1 U/mL IFNγ condition versus LFC of 1 U/mL IFNγ + norovirus condition. For volcano plots, the average log2 fold change (LFC) of all sgRNAs for each gene is plotted against the −log10(*P* value) for each gene. Blue and red highlighted genes in (A) represent a STARS score with FDR < 0.01, and dark gray genes represent non-targeting guides. Values in (B–D) represent means ± SEM from 2 to 3 independent experiments. *P ≤ 0.05, **P ≤ 0.01, ***P ≤ 0.001 and ****P ≤ 0.0001 were considered statistically significant. ns, not significant. *P* value was determined by two-way ANOVA with Dunnett’s multiple comparison test.

Guides for 18 genes (Fig. 3A; Table S4) were enriched in IFNγ-treated and norovirus-infected cells compared to mock conditions. We observed intermediate agreement of gene-level results, with a Pearson’s correlation of 0.47 between 1 U/mL IFNγ treatment alone compared to 1 U/mL IFNγ + norovirus-infected cells (Fig. 3B). Among the targeted genes were *G3bp1*, *Sptcl1*, and *Sptcl2* which are required for efficient norovirus replication (22, 51–53). Among the genes identified as candidates for enhancing IFNγ-induced inhibition of norovirus replication were *Iqgap1* and *Ufc1* (Fig. 3A; Table S4). Deficiency of IQGAP1 in human monocytic cells results in hyperactive type I IFN responses to cytosolic nucleotides (54). *Ufc1* and *Uba5* encode enzymes in the ubiquitin-like system that covalently conjugates UFM1 to target proteins (UFMylation herein) (27, 55–57). UFMylation is required to maintain ER homeostasis, so in the absence of UFMylation consequent ER stress can lead to overactivation of IFNγ responses (27). We therefore quantified the effects of IFNγ on norovirus replication in cells lacking *Ufc1* or *Uba5* (*Ufc1^−/−^*, *Uba5^−/−^*) (27). IFNγ more potently inhibited norovirus replication in these cells (Fig. 3C and D) as shown by diminished potency of IFNγ upon expression of wild-type proteins (*Ufc1*^WT^ or *Uba5*^WT^) but not the enzymatically dead versions of these proteins (*Ufc1*^ΔC116A^, *Uba5*^ΔC248A^) (Fig. 3C and D) (27). UBA5 contains a UFM1 interaction motif disrupted by the mutations of W340A/L344A (Fig. 3D, *Uba5*^ΔUFIM^) (27, 55, 56). Disruption of this motif prevented UBA5 from rescuing the increased IFNγ potency observed in *Uba5^−/−^* cells (Fig. 3C). However, deletion of UFM1 itself had no effect on IFNγ-induced control of norovirus infection (Fig. S4). As observed by Balce et al., *Ern1* deletion reversed effects on IFNγ potency observed in *Uba5^−/−^* cells, consistent with a role for this aspect of the ER stress response in modulating IFNγ action (Fig. 3E) (27).

### Identification of candidate genes required for efficient inhibition of norovirus-induced cytopathicity by IFNγ

**Fig 4:**
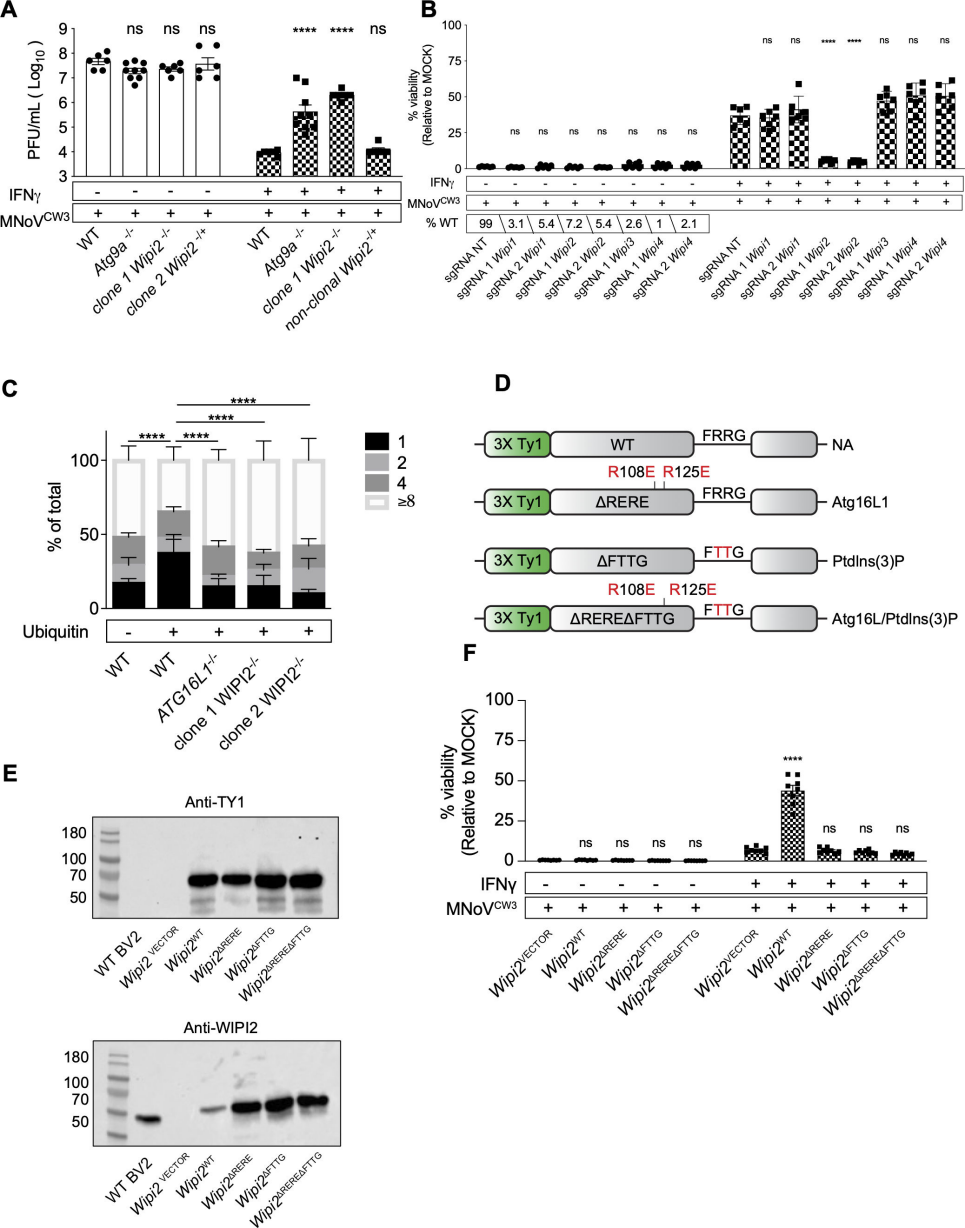
ATG16L1 and PtdIns(3)P-binding domains of WIPI2B are required for IFNγ-induced inhibition of norovirus cytopathicity in BV-2 cells; *WIPI2* is required for IFNγ-induced growth restriction of *T. gondii* in HeLa cells. (A) Plaque assay of WT, *Atg9a*^−/−^, *Wipi2*^−/−^ clonal, and Wipi2^−/+^ non-clonal knock-out cells as described in Fig. S1. (B) Viability assay of non-clonal BV-2-Cas9 knock-out cells transduced with non-targeting (NT) sgRNAs or sgRNAs targeting *Wipi1*, *Wipi2*, *Wipi3*, or *Wipi4 as described in Fig. S1*; percentage of the wild-type allele present in each non-clonal cell pool indicated. (C) *WIPI2^−/−^* HeLa cells were treated with IFNγ and infected with *T. gondii*. Parasites within ubiquitin positive (+) and ubiquitin negative (−) vacuoles were counted by immunofluorescence microscopy. (D) Schematic representation of WIPI2B complementation in clone 1 *Wipi2*^−/−^ BV-2 cells. (E) Western blot detection of TY1 epitope or endogenous WIPI2 in complemented cells. (F) Viability assay of *Wipi2*^VECTOR^, *Wipi2*^WT^, *Wipi2*^ΔRERE^, *Wipi2* ^ΔRERE^, and *Wipi2*^ΔRERE ΔFTTG^ complemented BV-2 cells as described in Fig. S1. Values in (A), (B), and (E) represent means ± SEM from 2 to 3 independent experiments. *P ≤ 0.05, **P ≤ 0.01, ***P ≤ 0.001, and ****P ≤ 0.0001 were considered statistically significant. ns, not significant. *P* value was determined by two-way ANOVA with Dunnett’s multiple comparison test; for *T. gondii* assay, *P* value was determined by two-way ANOVA with Tukey’s multiple comparison test.

We next compared guide frequencies between mock and 1 U/mL IFNγ + norovirus infection, reasoning that guides decreased in cells surviving norovirus infection of IFNγ-treated cells might have a role in IFNγ-induced inhibition of norovirus replication (Fig. 3A). Guides for 32 genes (Fig. 3A; Table S4) were decreased in frequency in IFNγ + norovirus-infected cells. Among these, the autophagy-related genes *Wipi2*, *Atg9a* as well as the ubiquitin ligase *Cul3* (below) were identified as candidates for a role in IFNγ-induced *ATG* gene-dependent innate immunity. To confirm screen findings, we generated one clonal BV-2 cell line lacking *Wipi2* (*Wipi2*^−/−^) (Table S1) and one non-clonal cell line lacking *Wipi2* (*Wipi2*^−/+^; cell line 2) (XX targeting of *Wipi2*, Table S1). We also examined BV-2 cells lacking *Atg9a* (*Atg9a*^−/−^) (Table S1) (27). *Wipi2* and *Atg9a* were required for efficient IFNγ-induced inhibition of norovirus replication (Fig. 4A), indicating that they constitute key components of this form of intracellular innate immunity. This contrasts with a recent report that WIPI2 is not required for IFNγ-induced inhibition of norovirus replication in BV-2 cells (32). While we cannot explain this difference, we report results from multiple different methods to inhibit Wipi2 expression herein (Fig. 4A and B) and performed our studies using 1 U/mL, a non-toxic dose (28, 34) rather than 100 U/mL IFNγ.

To understand the possible specificity of Wipi2, as compared to related family members, in IFNγ-induced control of norovirus infection, we used two independent guide RNAs to generate non-clonal cell lines targeting each *Wipi* family member [greater than 90% of alleles targeted (Table S1), Fig. 4B]. IFNγ inhibited norovirus infection in *Wipi1*, *Wipi3*, and *Wipi4* but not *Wipi2*, mutant cell lines. To determine the generality of the observation that *WIPI2* plays a key role in IFNγ-induced intracellular immunity we examined control of *T. gondii* in two independent clonal cell lines lacking *WIPI2.* We found that *WIPI2* is essential for efficient IFNγ-induced control of *T. gondii* replication within the PV (Fig. 4C)*.*

### Domains of WIPI2B and ATG16L1 required for IFNγ-induced inhibition of norovirus replication

We used mutagenesis to analyze the role of WIPI2 in greater detail because it interacts with ATG16L1 (58, 59), which plays a key role in IFNγ-induced inhibition of norovirus replication (Fig. 4D) (12, 17). Furthermore, it has been reported that the interaction between ATG16L1 and WIPI2 is important for the localization of LC3B to the norovirus replication complex in Hela cells (32). We defined the effects of the R108E/R125E (ΔRERE) mutations that inhibit binding to ATG16L1, the R224T/R225T (ΔFTTG) mutations that inhibit binding to PtdIns(3)P, and the double mutant ΔRERE/ΔFTTG (Fig. 4D) (58). We stably expressed these proteins and wild-type WIPI2B in *Wipi2*^−/−^ clone 1 cells and assessed protein expression by western blot analysis (Fig. 4E). Probing for an N-terminal epitope tag or endogenous WIPI2 revealed equivalent expression of the different constructs (Fig. 4E). Expression of WIPI2B rescued the capacity of IFNγ to inhibit norovirus infection in *Wipi2*^−/−^ cells (Fig. 4F). This confirms by complementation that the phenotype in this cell line is due to a lack of WIPI2B. In contrast, neither WIPI2B^ΔRERE^ nor WIPI2B^DFTTG^ rescued inhibition of norovirus after IFNγ treatment (Fig. 4F). The double mutant WIPI2B^DREREDFTTG^ protein also failed to restore IFNγ activity (Fig. 4F). Thus, WIPI2B binding to both ATG16L1 and PtdIns(3)P was necessary for immune control of norovirus by IFNγ.

**Fig 5:**
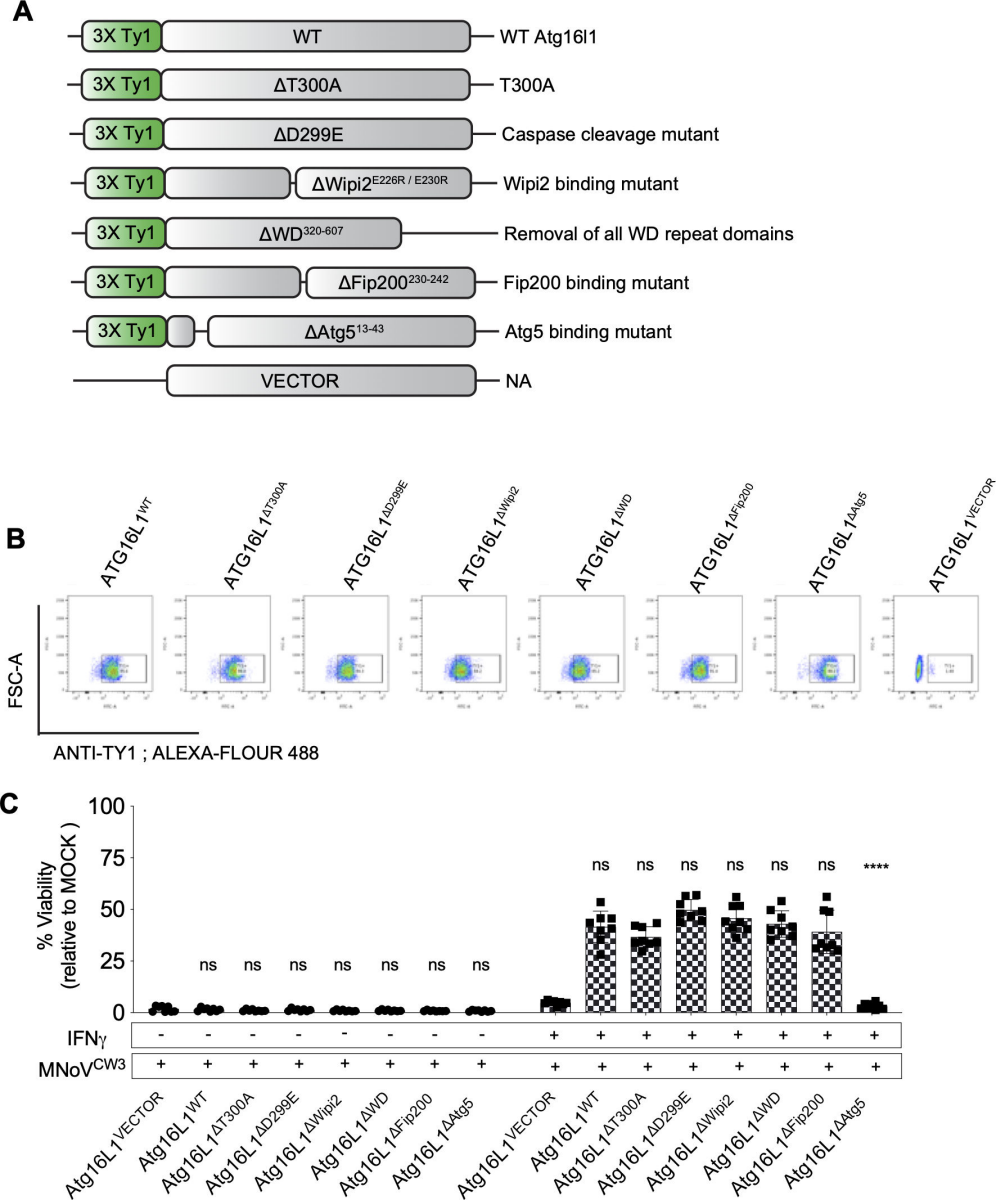
ATG5-binding domain of ATG16L1 is required for IFNγ-induced restriction of norovirus replication in BV-2 cells. (A) Schematic representation of ATG16L1 complementation in clone 1 *Atg16l1*^−/−^ BV-2 cells. (B) Intracellular staining and detection of TY1 epitope in complemented cells by flow cytometry. (C) Viability assay of *Atg16l1*^VECTOR^, *Atg16l1*^WT^, *Atg16l1*^ΔT300A^, *Atg16l1*^ΔD299E^, *Atg16l1*^ΔWipi2^, *Atg16l1*^ΔWD^, *Atg16l1*^ΔFip200^, *Atg16l1*^ΔAtg5^, and *Atg16l1*^VECTOR^ complemented BV-2 cells as described in Fig. S1. Values in (C) represent means ± SEM from three independent experiments. *P ≤ 0.05, **P ≤ 0.01, ***P ≤ 0.001, and ****P ≤ 0.0001 were considered statistically significant. ns, not significant. *P* value was determined by two-way ANOVA with Dunnett’s multiple comparison test.

As WIPI2 binding to ATG16L1 was implicated in IFNγ control of norovirus infection, we mutagenized amino acids with known functions in ATG16L1 and expressed them in ATG16L1-deficient cells (Fig. 1C, Fig. 5A). The resulting mutant proteins were efficiently expressed (Fig. 5B). This analysis revealed that, uniquely among these known ATG16L1 functions, binding to ATG5 is important in control of norovirus infection (Fig. 5C). This interaction is likely of functional importance because mutation of ATG5 so that it cannot conjugate to ATG12 interferes with this form of intracellular immunity (12, 28).

### *P62* is required for IFNγ-induced inhibition of *T. gondii* in human HeLa cells but not required for IFNγ-induced inhibition of norovirus replication in BV-2 cells

Many functions of autophagy and *ATG* genes involve the P62 protein. In murine cells**,** the ubiquitin-binding protein P62 is recruited to vacuoles containing *T. gondii* in an IFNγ-dependent manner but is ultimately dispensable for inhibiting replication of the parasite (60). In human cells, P62 is also recruited to parasitophorous vacuoles in an IFNγ-dependent manner (16) but its requirement for the restriction of *T. gondii* replication is unknown. Therefore, we generated clonal *P62*^−/−^ HeLa cells (Table S1) and observed that *P62* was required for IFNγ-induced inhibition of *T. gondii* (Fig. S5A) but was not required for IFNγ-induced inhibition of norovirus replication in clonal p62^−/−^ BV-2 cells (Fig. S5B; Table S1). Taken together, these data suggest the requirement for *P62* in IFNγ-mediated control of intracellular pathogens can be species or cell type-specific.

### *Cul3* and *Klhl9* are required for IFNγ-induced inhibition of norovirus replication

**Fig 6:**
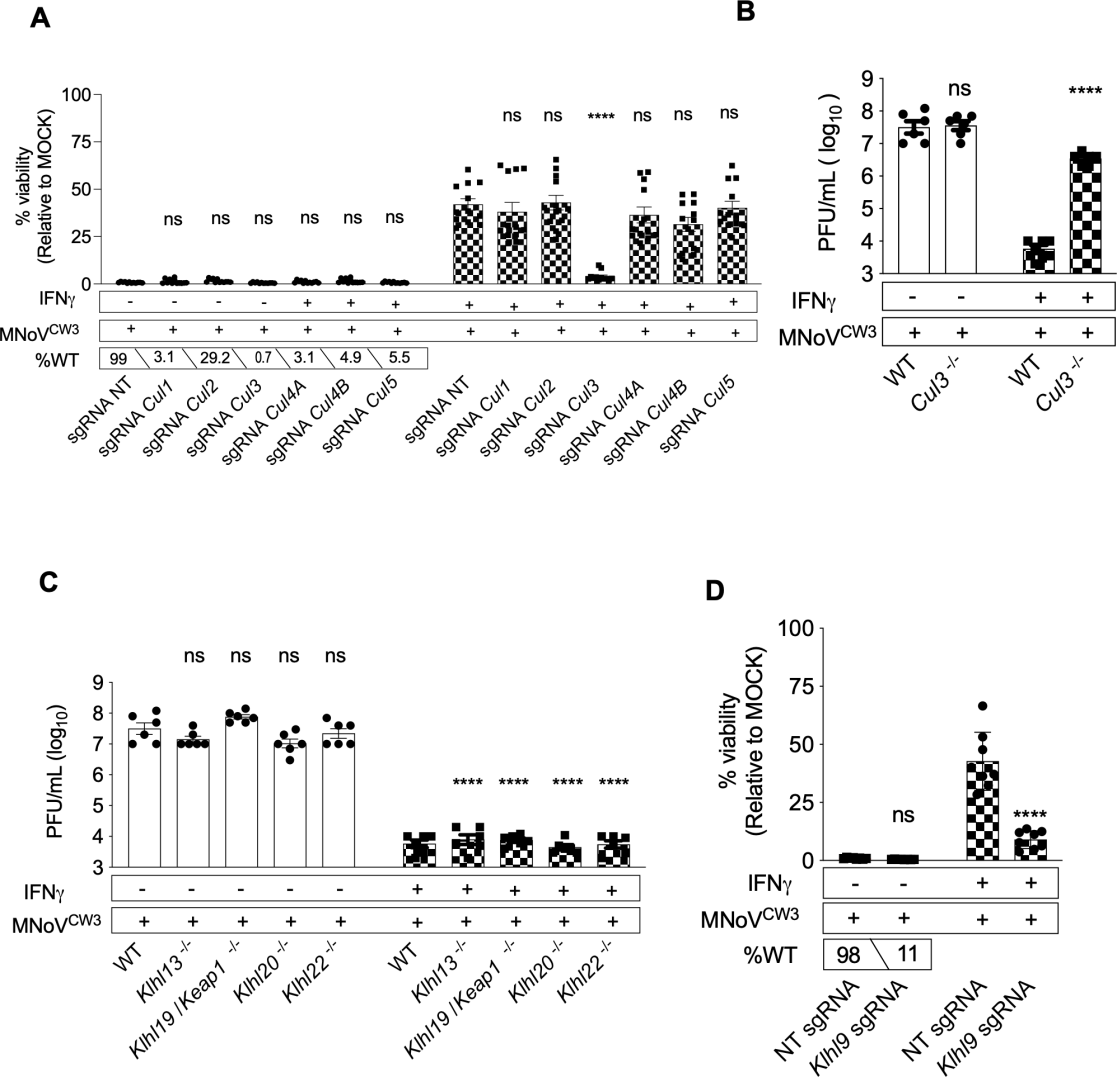
*Cul3* and *Klhl9* are required for IFNγ-induced inhibition of norovirus replication in BV-2 cells. (A) Viability assay of non-clonal BV-2-Cas9 knock-out cells transduced with non-targeting (NT) sgRNAs or sgRNAs targeting *Cul1*, *Cul2*, *Cul3*, *Cul4A*, *Cul4B*, and *Cul5 as described in Fig. S1*; percentage of the wild-type allele present in the non-clonal cell knock-out pool. (B and C) Plaque assay of WT, *Cul3*^−/−^, *Klhl13*^−/−^, *Klhl19*^−/−^, *Klhl20*^−/−^, and *Klhl22*^−/−^ BV-2 clonal knock-out cells as described in Fig. S1. (D) Viability assay of non-clonal BV-2-Cas9 knock-out cells transduced with NT sgRNA or sgRNA targeting *Klhl9 as described in Fig. S1*; percentage of the wild-type allele present in the non-clonal cell pool provided. Values in (A–D) represent means ± SEM from 2 to 3 independent experiments. *P ≤ 0.05, **P ≤ 0.01, ***P ≤ 0.001, and ****P ≤ 0.0001 were considered statistically significant. ns, not significant. *P* value was determined by two-way ANOVA with Dunnett’s multiple comparison test.

Given the important role of ubiquitin in autophagy, our attention was drawn to the screen finding suggesting that CUL3, a ubiquitin ligase involved in multiple cellular processes (61), was a candidate for a role in IFNγ-induced control of norovirus infection (Fig. 3A). We found that CRISPR guides targeting *Cul3* inhibited IFNγ-induced control of norovirus infection (Fig. 6A), a finding confirmed in clonal *Cul3^−/−^* cell line (Fig. 6B). In contrast, targeting *Cul1*, *Cul2*, *Cul4A*, *Cul4B*, and *Cul5* had no effect on control of norovirus infection (Fig. 6A). The specificity of CUL3-containing ubiquitin ligase complexes is conferred in some cases by assembly of CUL3 with various members of the KELCH domain family of proteins (61, 62). *KlhlL7* and *Klhl19* were the only KELCH domain protein-encoding genes included in our CRISPR library. Neither exhibited a meaningful effect on IFNγ-induced control of norovirus infection (Fig. 3A; Table S4). We therefore took a candidate approach to define a possible role for KELCH domain proteins in this form of intracellular immunity. Among KELCH domain proteins KLHL13 (63), KLHL19 (also Keap1) (64), KLHL20 (65), KLHL22 (66), and KLHL9 (63) are involved in cellular processes potentially related to the function of autophagy or *ATG* genes. Stable cell lines lacking *Klhl13*, *Klhl19/Keap1*, *Klhl20*, or *Klhl22* were all able to control norovirus infection in the presence of IFNγ (Fig. 6C). We were unable to generate a clonal cell line mutated for *Klhl9* (not shown). However, using *Klhl9* specific guides we generated non-clonal cell populations in which ~88% of the *Klhl9* alleles were disrupted (Fig. 6D). These cells were unable to effectively control norovirus infection in the presence of IFNγ, indicating that this KELCH domain protein is a candidate for involvement with CUL3 in IFNγ-induced control of norovirus infection.

## DISCUSSION

Intracellular pathogens present diverse challenges to the immune system because they hijack different aspects of cell biology to their advantage. IFNγ is a central mediator of innate and adaptive immunity, in part via orchestrating the activities of populations of immune cells. However, IFNγ also induces cell-intrinsic immunity to intracellular pathogens via induction of transcriptional and other cellular pathways that create a cellular environment that is hostile to invading pathogens. Understanding the molecular and cellular mechanisms for this process is important for defining mechanisms of immunity to infection. We report herein cellular genes that regulate intracellular immunity and identify new components of the autophagy machinery including *Gate16/GabarapL2*, *Atg9a*, and *Wipi2b* that play an autophagy-independent role in IFNγ-induced intracellular immunity to *T. gondii* and/or norovirus. In addition, we found a previously unsuspected role of *Cul3* and *Klhl9*, two components of a ubiquitin ligase complex, in this form of intracellular immunity. These data build on previous work on other *ATG* genes to elucidate a pathway that targets diverse pathogen-driven changes in intracellular membranes but that is not canonical degradative autophagy (11–19). The striking specificity of the genes *Wipi2*, *Cul3*, *GATE-16*, and *Klhl9* within their respective large gene families for this form of immunity supports the previously proposed concept that cassettes of specific genes, some of which are also involved in degradative autophagy, provide a highly sophisticated programmable armamentarium for intracellular immunity (11, 14, 17).

### *ATG* gene-dependent intracellular immunity

We and others have shown that IFNγ-induced intracellular immunity to norovirus and *T. gondii* does not require autophagy as a degradative function, but nevertheless requires essential *ATG* genes involved in the ubiquitin-like systems that conjugate ATG8 family proteins to PE during autophagy (11–14, 16, 17, 19, 67). The involved proteins include ATG7, which triggers the lipidation of ATG8 family proteins, ATG3 which complexes with ATG8 family proteins to foster their lipidation, and the E3-ligase-like ATG5-12-16L1 complex (11–14, 16, 17, 19, 67), which directs lipidation of ATG8 family proteins to appropriate sub-cellular locations. The ATG8 family comprises LC3 and GABARAP proteins involved in various aspects of autophagy and non-autophagic cellular processes (1, 4, 68). To further define the genetic requirements for IFNγ-induced inhibition of norovirus infection, we first validated the BV-2 cell system by demonstrating the expected roles for *Stat1*, *Irf1*, *Atg5*, and *Ag16L1* (12, 17, 24, 26). CRISPR screening in mock- or IFNγ-treated cells revealed genes essential for cell survival in the absence of viral infection; these were not studied further. Analysis of cells treated with IFNγ and infected with norovirus also revealed genes known to be essential for efficient norovirus replication (22, 51–53) and genes that regulate responses to IFNγ such as *Ufc1* and *Uba5*. In addition, this screen revealed new players in intracellular immunity including a single member of the murine ATG8 gene family. We found that GATE-16 is required for IFNγ-induced immunity to two very different intracellular pathogens in norovirus and *T. gondii*. This confirms and extends data indicating the murine GABARAPs, but not LC3 proteins, are required for IFNγ-induced intracellular immunity to *T. gondii* (18). The observation herein that a single ATG8 family member, GATE-16, is required for IFNγ to inhibit norovirus replication in this system is important for future studies since many studies use localization of LC3B as a marker for recruitment of ATG8 family proteins to autophagosomes or the norovirus replication complex. It may be that the mechanisms of recruitment to membranes are the same for all ATG8 family members, but in our system, only recruitment of GATE-16 is mechanistically important for cytokine control of viral replication.

It is notable that the cellular membranes targeted by these *ATG* genes are quite distinct for *T. gondii* and norovirus. *T. gondii* survives and replicates via binary fission inside a single membrane-bound vacuole created when it invades the cell. Replication occurs sequestered away from the cytoplasm. In contrast, norovirus replication occurs on the cytoplasmic face of intracellular membranes rearranged into a topologically complex replication complex responsible for orienting viral proteins and nucleic acids for efficient replication and assembly. Nevertheless, these two topologically distinct processes are interdicted by IFNγ in a manner dependent on a common set of *ATG* genes.

### Not all essential *ATG* genes are required for this form of intracellular immunity

The *ATG* genes required for this form of IFNγ-induced intracellular immunity provide clues as to the mechanisms involved, but so do the *ATG* genes that are not required. We confirmed the previously reported lack of a role for *Atg14* in IFNγ-induced inhibition of norovirus replication (17). This is a key observation because ATG14 is an essential component of the C1 form of the hetero-tetrameric Class III PI3Kinase complex required for the initiation of degradative autophagy (1, 3). Furthermore, ATG14 is not required for control of *T. gondii* in IFNγ-treated human HeLa cells (16). The C1 complex comprises ATG14, VPS15, VPS34, and Beclin 1, while the C2 complex substitutes UVRAG for ATG14 (1, 4, 5). We also show herein that UVRAG is not required for this form of intracellular immunity. These lipid kinase complexes generate PtdIns(3)P on membranes including the isolation membrane to initiate degradative autophagy. Beclin 1 is a core member of both autophagy-related Class III PI3Kinases. We report here that the *Becn1* gene was not required for IFNγ-mediated inhibition of norovirus replication. This confirms that the autophagy-related complexes C1 and C2 are not required for this form of intracellular immunity. This conclusion is supported by the observation that the same cell lines used here were used to show that *Becn1* and *Atg14* are required for protection against IFNγ-induced cell death in BV-2 cells (28). Thus, these genes are functional in this cellular system via their expected role in autophagy. It is notable that there is overlap between the genes required to prevent cell death induced by IFNγ, and those required for IFNγ to inhibit norovirus replication. For example, both require *Atg5* and *Atg7*. However, the mechanisms of IFNγ-induced inhibition of norovirus replication and prevention of cell death likely differ because prevention of IFNγ-induced cell death requires *Atg14* and *Becn1* (28), while prevention of norovirus replication does not. Together, these data show that the classical PI3K complexes involved in autophagy are not required for this form of intracellular immunity, despite the shared involvement of specific essential *ATG* genes that act downstream of the C1 and C2 complexes in degradative autophagy

The mechanism by which *ATG* proteins inhibit replication of both norovirus and *T. gondii* in IFNγ-treated cells involves the recruitment of IFN-inducible GTPases to the intracellular membranes responsible for supporting pathogen replication (15, 17, 67, 69–71). While the genes encoding sub-families of the interferon-γ-inducible immunity-related GTPase (IRGs) differ between humans and mice (67, 72), IRGs and guanylate-binding proteins (GBPs) in mice, and GBPs in humans (32, 73) are required for IFNγ-induced inhibition of the replication of both norovirus and *T. gondii*, as well as *Chlamydia* (71, 74, 75). In contrast to species variations in IFN-induced GTPases that act downstream of *ATG* proteins, the role of the ATG5-12-16L1 complex is conserved between humans and mice for this form of IFNγ-induced intracellular immunity. We speculate that there are conserved sequential events that result in targeting norovirus and *T. gondii*-related cellular membranes despite the striking differences in topology between the cellular compartments used by these two pathogens. First, the ATG5-12-16L1 complex is recruited to the relevant membranes followed by deposition of lipidated forms of the ATG8 family proteins. This is followed by recruitment of IFN-inducible GTPases to the target membranes [reviewed in reference (71)]. Less well-defined are events prior to ATG5-12-16L1 recruitment and those that occur after recruitment of IFNγ-induced immune effectors to pathogen-related membranes (71).

### Role of *Atg9a* in IFNγ-induced *ATG* gene-dependent immunity

*Atg9a* is a lipid scramblase (76) that plays its role in autophagy via provision of lipids to developing autophagosomes via recruitment of ATG9A-positive small vesicles (58, 68, 77). While the role of *Atg9a* demonstrated herein could well involve these small vesicles, the regulation of *Atg9a* in IFNγ-induced intracellular immunity appears to differ from its regulation during autophagosome formation. In murine cells, neither the unc-5-like kinases ULK1 nor ULK2, which are required for initiation of autophagy, are required for IFNγ-mediated inhibition of norovirus replication (17, 68). This is particularly interesting since the role of ATG9A trafficking in degradative autophagy is tightly regulated by ULK1 kinase (68). This suggests that the regulation of *Atg9a* function in IFNγ-induced *ATG* gene-dependent immunity to norovirus is likely via a regulatory cascade distinct from that utilized to regulate the role of ATG9 in autophagy. Furthermore, recent work supports a role for an ATG9/ATG2 complex in autophagy (76), but we did not find a signal in our CRISPR screen for either *Atg2a* or *Atg2*b in our search for genes involved in the control of norovirus infection (Table S4). Lack of a signal in a genetic screen is suggestive only, and so this point will require further investigation.

Our data that *Atg9a* plays a key role in IFNγ-mediated inhibition of norovirus infection may be explained by the finding that deletion of ATG9 mRNA enhances induction of iNOS protein expression by IFNγ (27). Thus, the role of *Atg9a* might be either direct through action of this protein on pathogen-related intracellular membranes or indirect through changes in IFNγ signaling, or both. Our findings of a negative regulatory role for the UFMylation activity of the UFC1 and UBA5 enzymes are likely due to such an indirect effect on IFNγ signaling, mechanistically through the enhancement of IFNγ response via *Ern1*-dependent ER stress responses that occur when UFMylation is inhibited (27).

### Role of WIPI2B in IFNγ-induced *ATG* gene-dependent immunity

The role of *Wipi2b* in our system may inform how the ATG5-12-16L1 complex is recruited to intracellular membranes during IFNγ-induced *ATG* gene-dependent intracellular immunity. WIPI2B binds to both PtdIns(3)P in cellular membranes and to ATG16L1 and is important for intracellular targeting and clearance of *Salmonella enterica serovar* Typhimurium via the recruitment of the Atg5-12-16L1 complex to cellular membranes followed by LC3 lipidation and the formation of autophagosomal membranes to engulf the bacteria (58). We found that amino acids required for WIPI2B to interact with both ATG16L1 and PtdIns(3)P were required for efficient IFNγ-induced inhibition of norovirus replication. One explanation for these findings is that WIPI2B is upstream of the effects of the ATG5-12-16L1 complex, perhaps by binding cellular lipids on target membranes and then recruitment of the ATG5-12-16L1 complex via binding to ATG16L1 as observed for its role in degradative autophagy (58). This concept is consistent with recent work showing that amino acids in ATG16L1 that confer binding of ATG16L1 to WIPI2B are important for IFNγ to inhibit norovirus replication (32). In contrast, we did not detect an effect of point mutations in murine ATG16L1 that inhibit binding of ATG6L1 to WIPI2B (58) on this form of immunity. These differences may relate to the fact that mutations used in the two studies differ in both types and in whether mutations were made in the full length versus a truncated form of ATG16L1 lacking the WD repeat region, or the possibility that the point mutations chosen here do not fully block binding of ATG16L1 to WIPI2B in the complex milieu of the viral replication complex. Further studies will be required to resolve this issue.

The finding herein that the PI3P-binding region of WIPI2B is important for IFNγ inhibition of norovirus replication presents an interesting conundrum because we found that *Becn1*, *Atg14*, and *Uvrag*, encoding proteins that are essential components of the Class III PI3Kinases to generate PtdIns(3)P required for autophagy, are not required for IFNγ-induced *ATG* gene-dependent inhibition of norovirus replication. Thus, the source of phospholipids that interact with WIPI2B in this form of immunity remains mysterious. Interestingly, activation of Beclin 1-dependent processes is not sufficient to target the parasitophorous vacuole inhabited by *T. gondii* for *ATG* gene-dependent immunity (16). It is interesting to speculate that this form of immunity may involve Beclin 2 as opposed to Beclin 1 (78–80). These observations open new questions on how pathogen-related membranes become labeled with specific phospholipids after IFNγ treatment. Resolving this question will require additional experimentation.

### Species specificity of ATG gene-dependent immunity

Intriguingly, *Atg9a* and *p62* were previously found to be dispensable for the localization of *p62*, GBPs, *Irga6*, and ubiquitin to the parasitophorous vacuole of *T. gondii* in mouse embryonic fibroblast cells (18, 60). However, we found herein that *P62* was required for IFNγ-induced inhibition of *T. gondii* in HeLa cells, but not for norovirus replication in murine cells. Thus, while there are shared *ATG* genes involved in IFNγ-induced immunity to these two pathogens, there are proteins such as ATG9A and P62, which may play pathogen- or cell-type-specific roles in these topologically distinct cellular compartments in different experimental systems.

### Role of CUL3 and KLHL9 in IFNγ-induced *ATG* gene-dependent immunity to norovirus infection

While the above studies shed light on some components of the pathway by which IFNγ controls intracellular pathogens, the mechanisms by which this process links to ubiquitin ligases have not been previously described. This is an important observation because the role of ATG8 family proteins, such as GATE-16 which we demonstrate herein is selectively required for this form of intracellular immunity, frequently requires ubiquitin ligase function. In this regard, we found that *Cul3*, but not other *Cul* gene family genes, plays a required role in this form of immunity to norovirus infection. The CUL proteins form ubiquitin ligases whose specificity is often conferred by the presence of specific KELCH domain-containing proteins in CUL protein-containing E3 ligase complexes. Based on our CRISPR screen data and subsequent confirmatory experiments, we found a role for KLHL9, leading us to speculate that the specificity of IFNγ-induced *ATG* gene-dependent immunity may rest on the function of E3 ligases such as CUL3 combined with specific KELCH domain proteins. It is particularly interesting in this regard that KLHL9 also plays a role in intracellular immunity to salmonella (63). Together, these data suggest that an *ATG* gene-dependent programmable intracellular system involved in the control of multiple different pathogens might rely on CUL/KELCH domain proteins in a combinatorial manner.

### Summary

Data presented here, and substantial prior work, demonstrate the existence and physiological importance of a unique IFNγ-induced *ATG* gene-dependent process responsible for the creation of a cellular environment that is hostile to membrane-dependent replication of phylogenetically distinct pathogens. This mechanism appears programmable with the participation of a core machinery including WIPI2B, ATG9A, ATG7, ATG5-12-16L1, ATG3, and the ATG8 family member GATE-16 that is regulated by proteins in the UFMylation pathway and the involvement of specific ubiquitin ligases such as CUL3, and rendered specific by KELCH domain-containing proteins (12, 17, 71). These new data further support the previous conclusion that cassettes of *ATG* genes have been leveraged by the immune system to perform cytokine-induced tasks that block replication and clear pathogens (3, 11, 12). This system is programmable via the involvement of a variety of gene products and focuses on altered intracellular membranes created as diverse pathogens hijack and evade normal cellular functions. Since intracellular pathogens require rearrangement and hijacking of intracellular membranes as a lifestyle, this form of immunity is an important barrier to infectious diseases.

## MATERIALS AND METHODS

### Cells

BV-2, HEK293T, and HeLa cells were cultured in Dulbecco’s modified Eagle medium (Gibco) with 10% fetal bovine serum (FBS), and 1% HEPES. BV-2 Cas9 cell lines were generated using standard protocols (22). For selection, 5 µg/mL puromycin (Thermofisher) and 5 µg/mL blasticidin (Thermofisher) were added to BV-2 cells.

BV-2 and HeLa clonal knock-out cells were generated by transiently introducing Cas9 and gRNAs (Table S1) via nucleofection into wild type, and clones were screened for indels by sequencing the target region with Illumina MiSeq at approximately 500× coverage. Indel signature frequency was determined using an in-house algorithm (GEiC, Washington University School of Medicine, St. Louis, MO, USA). Non-clonal knock-out pools were generated in BV-2 Cas9 cells by transduction of lentivirus carrying sgRNA (Table S1) (21) followed by puromycin selection; 5 days post-puromycin selection indel frequency was determined by sequencing the target (Table S1).

For cDNA expression of ATG16L1 and WIPI2B, proteins cells were transduced with lentivirus carrying the gene of interest with an N-terminal 3× Ty1 tag (Table S5) on the pCDH-CMV-MCS-T2A-Puro backbone (CD522A-1, System Biosciences). cDNA expression of ATG proteins has been previously described (22, 27, 28).

### Viruses and viral assays

MNoV^CW3^ (GenBank accession no. EF014462.1, termed norovirus in the main text) was generated by transfecting a molecular clone (81) into HEK293T cells (P0 stock), which was passaged on BV-2 cells. After two passages, infected cells were frozen at −80°C and thawed, cleared of cellular debris and virus was concentrated by tangential flow filtration. For infection, WT or knockout BV-2 cells were seeded at 10^4^ cells/well of a 96-well plate. After 8 hours, cells were treated with 1 U/mL of IFNγ (BioLegend). After 16 hours, norovirus was added at a multiplicity of infction (MOI) of 5.0. Infected cells were harvested at 24 hpi and frozen at −80°C prior to plaque assay. For cell viability, CellTiter-Glo reagent (Promega) was added to wells of a 96-well plate, incubated for 10 minutes at room temperature, and then cellular ATP content was measured. Viral titers were determined in triplicate by plaque assay on BV-2 cells. BV-2 cells were seeded at 2 × 10^6^ cells/well of a six-well plate, and 24 hours later, 100 μl of 10-fold serially diluted samples was applied to each well for 1 hour with orbital rocking at room temperature. Viral inoculum was aspirated, and 2 mL of warmed MEM containing 10% FBS, 2 mM L-gluatmine, 10 mM HEPES, and 1% methylcellulose were added. Plates were incubated for 48–60 hours prior to visualization after staining with 0.2% crystal violet in 20% ethanol.

### Antibodies and western blots

Cell lysates were run under reducing conditions on an Any kD Mini-PROTEAN TGX Stain-Free Protein Gel (BioRad) and imaged using a ChemiDoc imaging system (Biorad). WIPI2B and ATG16L1 proteins were tagged with three copies of Ty1 on the N terminus. Anti-Ty1 antibody (ThermoFisher #MA5-23513) was used at 1:1,000. Anti-Wipi2 (ThermoFisher # PA5-78465) was used at 1:1,000. Anti-beta-actin (R&D Systems #MAB8929) was used at 1:10,000. Anti-Uvrag (Cell-Singaling Technologies #5320) was used at 1:100. Anti-mouse horseradish peroxidase (HRP) (Jackson Immuno Research Laboratories #315-035-003) was used at 1:10,000. Anti-rabbit HRP (Jackson Immuno Research Laboratories #111-035-003) was used at 1:10,000.

### Autophagy CRISPR-Cas9 subpool generation and screen

BV-2-Cas9 cells (1.4 × 10^7^) (22, 27, 28) were transduced with a lentivirus stock (transduction efficiency 40%) resulting in 6 × 10^6^ cells. After 36 hours, puromycin was added, and transduced cells were selected for 5 days. Five days later, 1 × 10^7^ cells were harvested, and DNA was for sequencing (QIAamp DNA Blood Midi Kit, Qiagen). For each experimental condition (mock, IFNγ, and IFNγ + norovirus), 1 × 10^7^ cells were seeded in a 15-cm^2^ dish. Eight hours later, cells were treated with media or 1 U/mL IFNγ. After 16 hours, cells were either mock infected or infected with norovirus at an MOI of 5.0. Cells were harvested 24 hours later for isolation of DNA for sequencing and assessment of cell viability (Trypan blue exclusion). Genomic DNA was sequenced and analyzed (22, 27, 28). Volcano plots were generated using the hypergeometric distribution method (https://github.com/mhegde/volcano_plots), and screen results were analyzed using STARS (https://portals.broadinstitute.org/gpp/public/software/stars).

### *T. gondii* growth restriction assay

HeLa cells were treated with 100 U/mL IFNγ for 24 hours, infected with tachyzoites, and washed 2 hours later to remove extracellular parasites. Cells were fixed in 4% formaldehyde 24 hours later. *T. gondii* was localized using antibody against the RH strain tachyzoites (81), and ubiquitin was localized using mouse antibody clone FK2 (04-263; EMD Millipore Corporation). Parasites were enumerated per PV from 30 PV on three individual coverslips from three independent experiments.

### Quantification and statistical analysis

Data were analyzed with Prism 7 software (GraphPad Software, San Diego, CA, USA). Volcano plot and Pearson visualizations were generated using R-studio (Integrated Development for R. RStudio, PBC, Boston, MA, USA; http://www.rstudio.com/).

## SUPPLEMENTAL MATERIAL

Fig. S1 mbio.02332-23-s0001.pdf Schematic for seeding, treatment, and norovirus infection.

Fig. S2 mbio.02332-23-s0002.pdf Autophagy CRISPR library screen design.

Fig. S3 mbio.02332-23-s0003.pdf Uvrag^-/-^ BV-2 cells lack UVRAG protein.

Fig. S4 mbio.02332-23-s0004.pdf Ufm1 is not required for IFNγ-induced inhibition of norovirus replication in BV-2 cells.

Fig. S5 mbio.02332-23-s0005.pdf p62 is not required for IFNγ-induced inhibition of norovirus replication in BV-2 cells; P62 is required for IFNγ-induced growth restriction of *T. gondii* in HeLa cells.

Table S1 mbio.02332-23-s0006.xlsx NGS validation data.

Table S2 mbio.02332-23-s0007.xlsx Autophagy CRISPR library.

Table S3 mbio.02332-23-s0008.xlsx Genes enriched and depleted for passage of BV-2 cells results; genes depleted during passage of BV-2 cells compared to Project Achilles.

Table S4 mbio.02332-23-s0009.xlsx Genes enriched and depleted in 1 U/mL IFNγ treated cells, 10 U/mL IFNγ treated cells, or 1 U/mL IFNγ + norovirus infected cells.

Table S5 mbio.02332-23-s0010.xlsx Supplemental sequences.

ASM does not own the copyrights to Supplemental Material that may be linked to, or accessed through, an article. The authors have granted ASM a non-exclusive, world-wide license to publish the Supplemental Material files. Please contact the corresponding author directly for reuse.
