# Supplementary material for: Autophagy gene-dependent intracellular immunity triggered by interferon-γ
Source: mBio. 2023 Oct 31;14(6):e02332-23. doi: 10.1128/mbio.02332-23 (PMC10746157; doi:10.1128/mbio.02332-23)
Supplement: Fig. S1 — Schematic for seeding, treatment, and norovirus infection. [file mbio.02332-23-s0001.pdf]

A

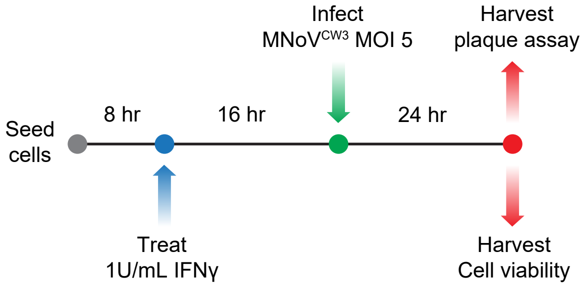

Figure S1. Schematic for seeding, treatment and norovirus infection.
Experimental workflow for IFNγ treatment and virus infection in BV-2 cells.
