# Supplementary material for: Autophagy gene-dependent intracellular immunity triggered by interferon-γ
Source: mBio. 2023 Oct 31;14(6):e02332-23. doi: 10.1128/mbio.02332-23 (PMC10746157; doi:10.1128/mbio.02332-23)
Supplement: Fig. S2 — Autophagy CRISPR library screen design. [file mbio.02332-23-s0002.pdf]

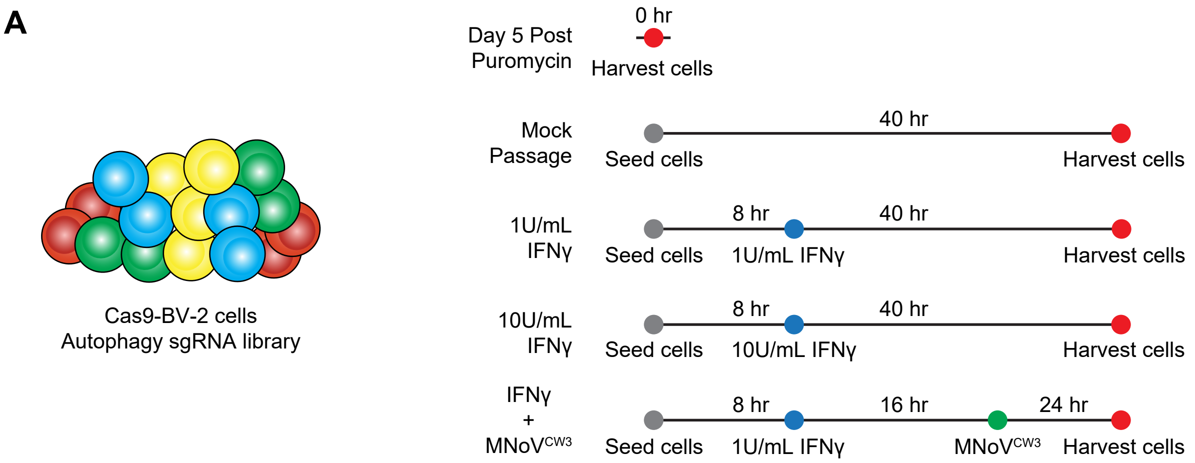

Figure S2. Autophagy CRISPR library screen design.
(A) BV2-Cas9 cells transduced with the Autophagy CRISPR library were collected for the following conditions: five days post puromycin selection, mock treatment, 1U/mL IFNγ treatment, 10U/mL IFNγ treatment or 1U/mL IFNγ treatment + $MNoV^{CW3}$.
