# Supplementary material for: Autophagy gene-dependent intracellular immunity triggered by interferon-γ
Source: mBio. 2023 Oct 31;14(6):e02332-23. doi: 10.1128/mbio.02332-23 (PMC10746157; doi:10.1128/mbio.02332-23)
Supplement: Fig. S3 — Uvrag-/- BV-2 cells lack UVRAG protein. [file mbio.02332-23-s0003.pdf]

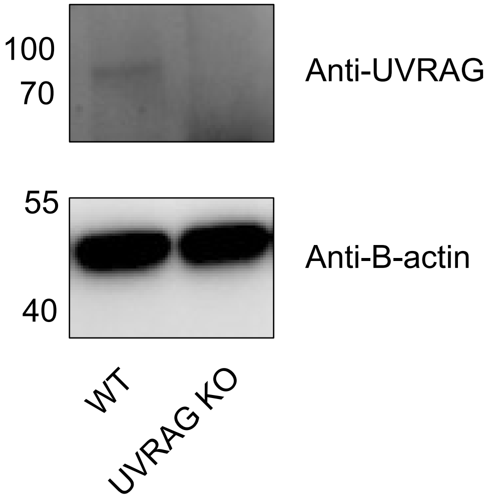

Figure S3. Uvrag-/- BV-2 cells lack UVRAG protein.
Western blot detection of endogenous UVRAG protein in WT or Uvrag-/- BV-2 cells, β-ACTIN was used as a protein loading control.
