# Supplementary material for: Autophagy gene-dependent intracellular immunity triggered by interferon-γ
Source: mBio. 2023 Oct 31;14(6):e02332-23. doi: 10.1128/mbio.02332-23 (PMC10746157; doi:10.1128/mbio.02332-23)
Supplement: Fig. S4 — Ufm1 is not required for IFNγ-induced inhibition of norovirus replication in BV-2 cells. [file mbio.02332-23-s0004.pdf]

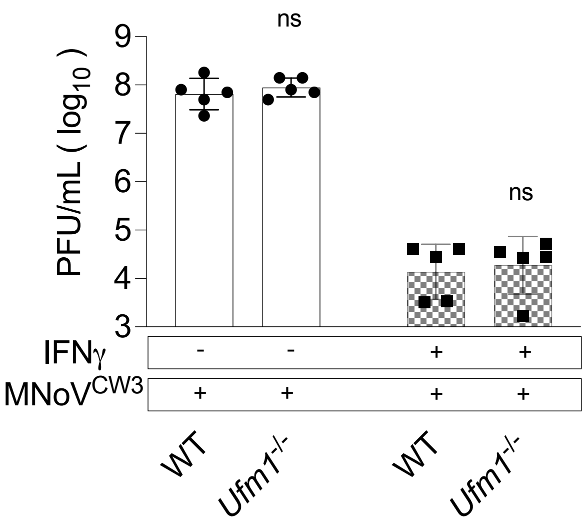

Figure S4. Ufm1 is not required for IFNγ-induced inhibition of norovirus replication in BV-2 cells.
Plaque assay of WT or Ufm1-/- BV-2 cells. Average data pooled from two independent experiments are represented as means ± SEM. P value ≤ 0.05 (*), ≤ 0.01(**), ≤0.001 (***), ≤ 0.0001 (****) were considered statistically significant. ns, not significant. P value determined by 2-way ANOVA with Dunnett's multiple comparison test.
