# Supplementary material for: Autophagy gene-dependent intracellular immunity triggered by interferon-γ
Source: mBio. 2023 Oct 31;14(6):e02332-23. doi: 10.1128/mbio.02332-23 (PMC10746157; doi:10.1128/mbio.02332-23)
Supplement: Fig. S5 — p62 is not required for IFNγ-induced inhibition of norovirus replication in BV-2 cells; P62 is required for IFNγ-induced growth restriction of T. gondii in HeLa cells. [file mbio.02332-23-s0005.pdf]

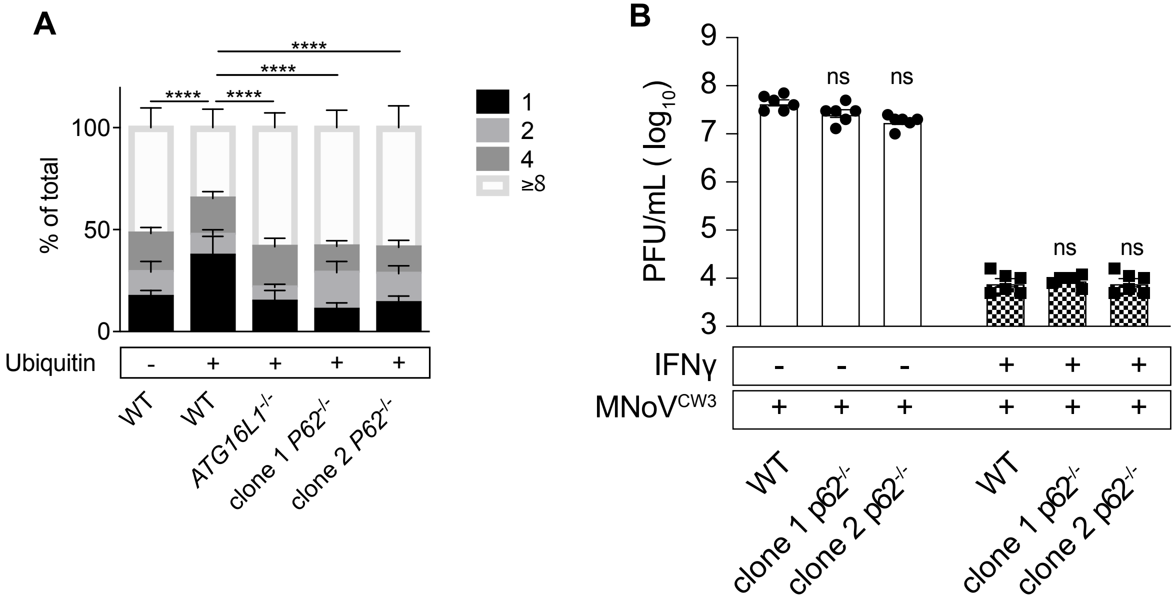

Figure S5. p62 is not required for IFNγ-induced inhibition of norovirus replication in BV-2 cells; P62 is required for IFNγ-induced growth restriction of T. gondii in HeLa cells. (A) P62-/- HeLa cells were treated with IFNγ and infected with T. gondii. Parasites within ubiquitin positive (+) and ubiquitin negative (-) vacuoles were counted by immunofluorescence microscopy. (B) Plaque assay of WT or p62-/- BV-2 cells. Average data pooled from 2-3 independent experiments are represented as means ± SEM. P value ≤ 0.05 (*), ≤ 0.01(**), ≤0.001 (***), ≤ 0.0001 (****) were considered statistically significant. ns, not significant. For T. gondii assay, P value determined by 2-way ANOVA with Tukey's multiple comparison test; for plaque assay P value determined by 2-way ANOVA with Dunnet's multiple comparison.
